# Dietary Resistant Potato Starch Alters Intestinal Microbial Communities and Their Metabolites, and Markers of Immune Regulation and Barrier Function in Swine

**DOI:** 10.3389/fimmu.2019.01381

**Published:** 2019-06-19

**Authors:** Julian Trachsel, Cassidy Briggs, Nicholas K. Gabler, Heather K. Allen, Crystal L. Loving

**Affiliations:** ^1^Food Safety and Enteric Pathogens Research Unit, National Animal Disease Center, Agricultural Research Service, Ames, IA, United States; ^2^Interdepartmental Microbiology Graduate Program, Iowa State University, Ames, IA, United States; ^3^Summer Scholar Research Program, College of Veterinary Medicine, Iowa State University, Ames, IA, United States; ^4^Department of Animal Science, Iowa State University, Ames, IA, United States

**Keywords:** resistant starch, mucosal barrier, T-regulatory cells, microbiota, SCFAs

## Abstract

Interactions between diet, the microbiota, and the host set the ecological conditions in the gut and have broad implications for health. Prebiotics are dietary compounds that may shift conditions toward health by promoting the growth of beneficial microbes that produce metabolites capable of modulating host cells. This study's objective was to assess how a dietary prebiotic could impact host tissues via modulation of the intestinal microbiota. Pigs fed a diet amended with 5% resistant potato starch (RPS) exhibited alterations associated with gut health relative to swine fed an unamended control diet (CON). RPS intake increased abundances of anaerobic *Clostridia* in feces and several tissues, as well as intestinal concentrations of butyrate. Functional gene amplicons suggested bacteria similar to *Anaerostipes hadrus* were stimulated by RPS intake. The CON treatment exhibited increased abundances of several genera of *Proteobacteria* (which utilize respiratory metabolisms) in several intestinal locations. RPS intake increased the abundance of regulatory T cells in the cecum, but not periphery, and cecal immune status alterations were indicative of enhanced mucosal defenses. A network analysis of host and microbial changes in the cecum revealed that regulatory T cells positively correlated with butyrate concentration, luminal IgA concentration, expression of IL-6 and DEF1B, and several mucosa-associated bacterial taxa. Thus, the administration of RPS modulated the microbiota and host immune status, altering markers of cecal barrier function and immunological tolerance, and suggesting a reduced niche for bacterial respiration.

## Introduction

Dietary prebiotics, such as resistant starches, provide an attractive alternative to sub-therapeutic antibiotics for improved animal health, and overall improved gut health in humans (1, 2). Resistant starches are compounds that are only minimally digested by the host and commensal microbes in the upper GI tract, thereby arriving in the large intestine as microbial-accessible carbohydrates (1). Bacteria that consume resistant starches are in a mutualistic relationship with the host, and as permanent residents, are associated with intestinal health (3). Fermentable carbohydrates are depleted as the digesta moves through the large intestine, consequently microbes often shift from utilizing diet-derived to host-derived carbohydrates. Without adequate access to diet-derived carbohydrates, bacteria will harvest host-derived sugars from the intestinal mucus layer. If the rate of utilization outpaces the rate of replenishment, barrier function can be compromised (4). However, if dietary carbohydrates are accessible, bacteria will ferment these compounds and release beneficial metabolites, particularly short-chain fatty acids (SCFAs). Host cells consume the vast majority of microbial-produced SCFAs, fueling intestinal homeostasis (5–7).

SCFAs are central metabolites for maintaining intestinal homeostasis. Butyrate in particular has a large body of work linking it to gut health, though other SCFAs such as propionate and valerate are recognized as important as well (8, 9). These metabolites have been shown to affect host tissues via G-protein coupled receptor signaling and inhibition of histone deacetylases (9, 10). When colonocytes oxidize SCFAs (such as butyrate) oxygen is consumed. This oxygen consumption lowers the oxygen potential of the epithelia, reducing the amount of electron acceptors available for bacterial respiration (6, 11–13). Furthermore, butyrate (and other SCFAs) can help to limit immune activation by enhancing mucosal barrier function and immunological tolerance, reducing the secretion of immune-derived reactive oxygen and nitrogen species, which also can be used in microbial respiration. SCFAs benefit mucosal barrier function by stimulating increased secretion of mucus, antimicrobial peptides (14), and IgA (7), therefore preventing the translocation of intestinal bacteria that would elicit an immune response. Second, SCFAs, such as butyrate, can induce a more tolerant immune phenotype through the generation of several regulatory immune cell types (15). In total, SCFA-driven changes to the gut microenvironment limit the niche for microbes with respiratory metabolisms (11, 12), allowing microbes that specialize in fermentation to outcompete those that respire, such as *Campylobacter, Salmonella*, and *Escherichia* species (6, 13).

Dietary intake of resistant starches may support a healthy intestinal ecosystem and limit the negative impact of weaning on mammalian health, though these effects can depend on the type of resistant starch consumed as well as the existing microbiota (16, 17). For example, feeding resistant potato starch (RPS) to nursery-aged piglets enhances some markers of gut health (18). However, the mechanisms by which RPS supports intestinal health in the weaned mammal are poorly defined. This study was designed to investigate how microbial changes during prebiotic consumption affect the weaned piglet's mucosal immune status. We chose to investigate the impacts of RPS, a type 2 resistant starch, at a 5% inclusion rate due to benefits seen at low inclusion rates (0.5 and 1%) (18) but some detrimental effects at a high inclusion rate (14%) (19). Based on these previous studies, and the larger body of evidence regarding prebiotics, we proceeded with the hypothesis that intake of this prebiotic would modulate the gut microbiota and their metabolic outputs and that these changes would benefit host tissues. The effects of prebiotics are mediated through the microbiota and occur mainly in the distal gut; therefore, the analysis was focused on the ileum, cecum, colon, and feces. Additionally, butyrate is a major metabolite of intestinal bacteria that can directly impact host cells; thus, *but* gene amplicons, a bacterial gene for butyrate production (20), were assessed to gain more detailed information on changes to the butyrate producing bacterial community and the expression of *but* genes. Combining the 16S and *but* amplicon datasets allowed simultaneous investigation of broader changes in the total bacterial community as well as more specific changes in a bacterial function of central importance in the gut.

## Materials and Methods

### Experimental Design

Ten pregnant, Large White crossbred sows were delivered 2 weeks prior to farrowing, and farrowed onsite. Base diets were formulated in accordance with industry standards including phase changes as the piglets aged. At 14 days-of-age, piglets were offered non-amended Phase 1 starter diet (Table S1). At 21 days-of-age, piglets were weaned, and separated into two treatment groups. Treatment groups consisted of two pens of seven piglets for a total of 14 piglets in each treatment group, each group had equal representation from all litters. The control group (CON) continued to receive non-amended Phase 1 Starter Diet. The treatment group was fed Phase 1 Starter Diet amended with 5% raw potato starch (RPS; MSP Starch Products Inc., Carberry, Manitoba, Canada, >70% resistant starch by dry weight, AOAC 2002.02 method). At 12 days post-weaning (33 days-of-age), the CON group was switched to non-amended Phase 2 Diet and the RPS group switched to Phase 2 Diet amended with 5% raw potato starch (Table S1). At 21 days post-weaning (42 days-of-age), seven piglets from each group (three from one pen and four from a second pen) were humanely euthanized by injection of sodium pentobarbital (Vortech Pharmaceuticals). All animal procedures were performed in compliance with the National Animal Disease Center Animal Care and Use Committee guidelines and review.

### Sample Collection

Piglets were weighed at weaning and necropsy. Fecal samples were collected at 0, 12, 15, 19, and 21 days post-weaning. Feces were collected fresh and transported on ice, aliquoted for downstream applications, and stored at −80°C. Prior to euthanasia, peripheral blood was collected into sodium citrate cell-preparation tubes and tubes transported to the laboratory at room temperature according to manufacturer's recommendations (BD Pharmingen). At necropsy, cecal contents were collected into RNALater and stored at 4°C until RNA extraction, and snap frozen and stored at −80°C. Sections of cecal tissue were gently rinsed with phosphate-buffered saline (PBS) and the mucosae were scraped with a sterile cell lifter. One portion of mucosal scrapings was immediately stored in RNALater at 4°C for host RNA extraction and another frozen at −80°C for bacterial DNA extraction. Ileocecal lymph node and sections of cecal tissue were collected in the appropriate buffer on ice and immediately processed for flow-cytometry.

### Immunohistochemical Staining (IHC)

Fresh cecal tissues were formalin-fixed and sections prepared using standard histological techniques. Details for CD3 and IgA staining are available in the supplement. Slides were scanned into Spectrum Version 11.2.0.780 (Aperio Technologies, Inc.) and Aperio ImageScope was used for annotation and to quantify cell populations. Cell counts were obtained using a nuclear algorithm on Aperio ScanScope software and are reported as cells/mm^2^.

### Phenotypic Analysis by Flow Cytometry

At necropsy, ~2 g of gently rinsed cecal tissue was placed in complete RPMI (RPMI 1640 [Life Technologies; Grand Island, NY] supplemented with 10% fetal calf serum [FCS, Omega Scientific; Tarzana, CA], L-glutamine [Life Technologies], 25 mM HEPES [Sigma; St. Louis, MO], essential amino acids and antibiotics [Sigma]) and stored on ice until processing. A previously described protocol was adapted for isolation of both epithelial cells and lamina propria cells from cecal tissue (21), with some modifications (Supplementary Information). Cells from peripheral blood and ileocecal lymph nodes were isolated as previously described (22). Approximately 10^6^ cells per tissue were used for flow cytometric analysis.

Cells were stained with Zombie Yellow Viability dye (Biolegend, San Diego, CA), followed by incubation with fluorescently-conjugated anti-porcine monoclonal antibodies [BD Biosciences, San Jose, CA (except as noted)]. Antibodies used included anti-porcine CD3 (clone BB23-8E6-8C6), CD4 (clone 74-12-4), CD8α (clone 76-2-11), CD25 (clone K231.3B2, Southern Biotech), γδTCR (clone MAC320), and FOXP3 (clone FJK16s). For staining of FoxP3 the Intracellular Nuclear Staining Kit (Biolegend) was used. Data were acquired on a BD LSRII instrument and analyzed with FlowJo Software (FlowJo LLC, Ashland, Oregon). Representative flow plots and gating strategy are available (Figures S1, S2).

### RNA Extraction, cDNA Synthesis, and RT-qPCR of Cecal Tissue

Host RNA was extracted using the TriReagent (Life Technologies)-modified protocol with the PowerLyzer UltraClean Tissue and Cells RNA Isolation Kit (MoBio Laboratories, Inc.). Homogenization in TriReagent was carried out in a Thermo Savant FastPrep^®^ FP120 Cell Disrupter (Qbiogene, Inc., Carlsbad, CA). An on-column DNAse step was included (On-Spin Column DNase I Kit, Mo Bio Laboratories, Inc.). RIN values were 5.5 or higher. QuantiTect Reverse Transcription Kit (Qiagen, Valencia, CA) was used for cDNA synthesis.

Gene expression was measured using the TaqMan^®^ Universal Master Mix II system (Applied Biosystems, Foster City, CA). Cycling conditions were 40 cycles of 95°C for 15 s and 60°C for 1 min. The gene β-actin was used to normalize the expression of target genes according to the 2^ΔΔCq^ method (23). Primers and probes are described in Table S3.

### Microbial Community Analysis

Microbial nucleic acids were extracted from feces and cecal contents using the PowerMag fecal DNA/RNA extraction kit (MoBio). Only cecal contents were used for RNA isolation. RNA samples were treated with DNase Max (MoBio) kit, and converted to cDNA using the High Capacity cDNA Synthesis Kit (Applied Biosystems). Amplicons of the V4 region were generated and sequenced in accordance with the protocol from Kozich et al. (24). Amplicons of the butyryl-CoA:acetate CoA transferase (*but*) gene were generated and sequenced using the protocol from Trachsel et al. (20). Both the 16S rRNA and *but* gene libraries were sequenced on a MiSeq (Illumina) using 2 ×250 V2 and 2 ×300 V3 chemistry, respectively.

### IgA Measurements

Cecal contents (~250 mg) were lyophilized, resuspended in extraction buffer (10 mM Tris, 100 mM NaCl, 1 mM CaCl_2_, 0.5% Tween-20, 1 tablet cOmplete™, EDTA-free Protease Inhibitor Cocktail (Roche, Branford, CT) per 100 mL) at 1 mL extraction buffer per 30 mg freeze-dried cecal contents, and vortexed on high for 10 min. Debris was pelleted by centrifugation at 5000× g and dilutions of the supernatant were used in the Pig IgA ELISA Quantitation kit (Bethyl Laboratoties, Montgomery, TX), and the final coefficient of variation (CV) for the IgA ELISA was 0.24. Results are reported as ng IgA/mg dry contents.

### Short-Chain Fatty Acid (SCFA) Measurements

One gram of material (cecal contents or feces) was suspended in 2 mL PBS, vortexed for 1 min, and debris was pelleted by centrifugation at 5000× g for 10 min. Supernatant (1 mL) was added to heptanoic acid internal standards. Butylated fatty acid esters were generated as described (25), and analyzed using an Agilent 7890 GC (Agilent, Santa Clara, CA). This assay measures the following SCFAs: formate, acetate, propionate, isobutyrate, butyrate, lactate, isovalerate, valerate, caproate, oxalate, phenylacetate, succinate, and fumarate. Total SCFAs are the sum of all compounds.

### Bioinformatics

Both the 16S rRNA gene and *but* gene amplicon data were clustered into operational taxonomic units (OTUs) with 97% similarity in mothur using the Miseq SOP (26). 16S rRNA gene sequences were aligned to the SILVA reference alignment, and *but* sequences were aligned to an alignment of *but* reference sequences downloaded from RDP's fungene database (27). Singletons and Doubletons were removed prior to distance matrix calculation and OTU clustering for both the 16S rRNA gene and *but* gene amplicons. Error rates calculated by sequencing mock communities (28) for 16S rRNA genes and *but* genes were 1.3e-06 and 2.2e-03 errors per basecall, respectively.

### Statistics

Unless otherwise stated, Wilcoxon tests were used to test for statistical differences because a robust determination of normality was not feasible. Unless explicitly stated, no outliers were removed from this study. The R package vegan (29) was used to carry out ecological analyses. Community structure similarity analyses were performed by calculating Bray-Curtis dissimilarities on rarefied OTU tables, and statistical testing was accomplished using vegan's adonis and betadisper functions. Differential abundance was determined using the DESeq2 package (30) using Wald tests with parametric fits and FDR corrected *P*-values. Prior to testing, OTUs with fewer than 10 counts globally were removed and the resulting unrarefied counts were used as the input for DESeq2 in accordance with the package recommendations. Correlations for network analysis were calculated with CCREPE (31) for compositional data or the rcorr function from the hmsic R package. Network layout was performed with the geomnet R package (32). Only significant, positive correlations with a Spearman coefficient of at least 0.6 are shown.

### Data Availability

Raw sequence data is available in the SRA under project accession number PRJNA476557. All processed data and R scripts used in this analysis are available at https://github.com/Jtrachsel/RPS-2017.

## Results

### Overt Differences in Health Were Not Observed Between the Two Treatment Groups

No obvious differences in health or behavior were observed between the groups. No significant differences in weights were observed either at weaning or necropsy. No gross pathology was observed at necropsy, nor were microscopic pathological changes noted in sections of cecal tissues. These data suggest that all animals in this study were healthy regardless of treatment.

### Bacterial Communities Differed Between the Two Treatment Groups

**Figure 1:**
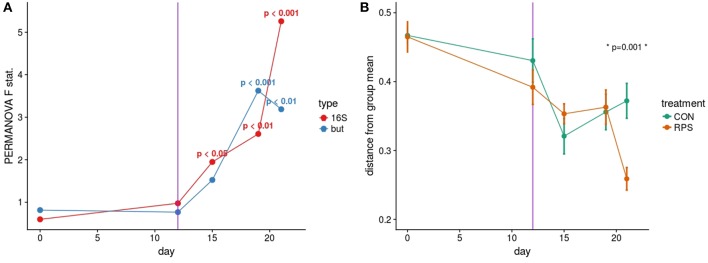
**(A)** PERMANOVA pseudo F statistic over time for both the 16S- and *but* gene-based fecal bacterial community analyses. A Bray-Curtis dissimilarity matrix was calculated from a rarefied set of OTUs clustered at 97% identity, and this dissimilarity matrix was used as the input for the PERMANOVA tests. Each data point represents the PERMANOVA test statistic (F: intergroup dissimilarity/intragroup dissimilarity) comparing the CON and RPS fecal bacterial community structure at each time point; higher values equate to greater differences between treatment groups. *P*-values are shown when *p* < 0.05. **(B)** Group dispersion over time. Higher values on the Y axis indicate that communities within that group are more dissimilar from each other. Lower Y axis values indicate that communities in that group are more similar to each other. Error bars represent the standard error around the mean. The vertical purple line indicates a dietary phase change.

Based on the PERMANOVA tests for community similarity, both the CON and RPS groups showed similar weaning-related changes in their bacterial communities; however, the communities of the groups became less similar over time. Group fecal bacterial community structures did not differ significantly until day 15, which coincided with a dietary phase change (12 days post-weaning), marked by a decrease in the amount of dietary lactose (Figure 1A; Table S4). By day 21 the two treatment groups had significantly different fecal bacterial community structures. Group differences in community structures were seen in both the 16S rRNA gene sequence-based analysis, as well as the *but*-based analysis. The size of these effects were driven in part by community dispersion (Figure 1B). The 16S rRNA gene-based analysis showed significantly less intra-group variability among the RPS-fed pigs compared to the CON-fed pigs at day 21, and the bacterial community structure of both groups became less dispersed as they matured (Figure 1B; Table S5). These results suggest that weaning-associated changes in bacterial communities were more profound than RPS-associated changes, and that RPS intake did not immediately affect the composition of the microbiota, but over time, significantly affected both the overall community composition and the intragroup variability as these communities matured.

**Figure 2:**
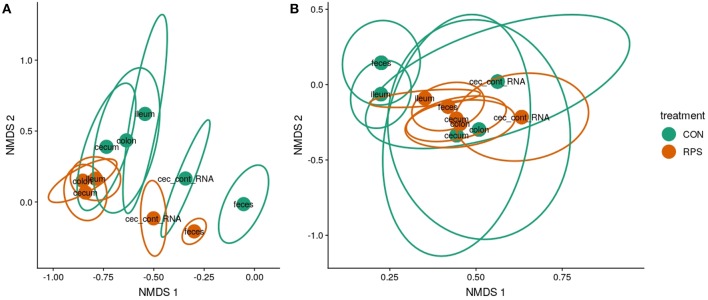
Bacterial community structure in RPS-fed pigs (orange) compared to CON-fed pigs (green) at day 21. Nonmetric multidimensional scaling (NMDS) ordinations of 16S rRNA gene-based Bray-Curtis dissimilarities calculated from rarefied OTU tables (clustered at 97% identity) **(A)** and *but* gene-based Bray-Curtis dissimilarities calculated from rarefied OTU tables (clustered at 97% identity). **(B)** Points indicate group centroids, from discrete gut locations as labeled. Centroids represent the approximate average community composition, and ellipses show standard error around this average. “Ileum” = ileal mucosa, “cecum” = cecal mucosa, “colon” = colonic mucosa and “Cec_cont_RNA” = RNA from cecal contents.

Twenty-one days after weaning, the structure of the microbiota was significantly different between the CON and RPS groups at multiple intestinal locations. The 16S rRNA gene-based analyses tended to show greater differences than the *but* gene-based analyses (Figures 2A,B; Table S4). The tissue-associated bacterial communities (ileal mucosa, cecal mucosa, colonic mucosa, and RNA from cecal contents) exhibited the same dispersion trend as the fecal communities, with the communities in tissues from RPS-fed pigs exhibiting less group dispersion (Table S5). These data suggest that the effects of RPS intake on bacterial communities were not limited to one tissue or location, but bacterial populations throughout the intestinal tract were similarly impacted resulting in higher intragroup similarity in the RPS animals.

**Figure 3:**
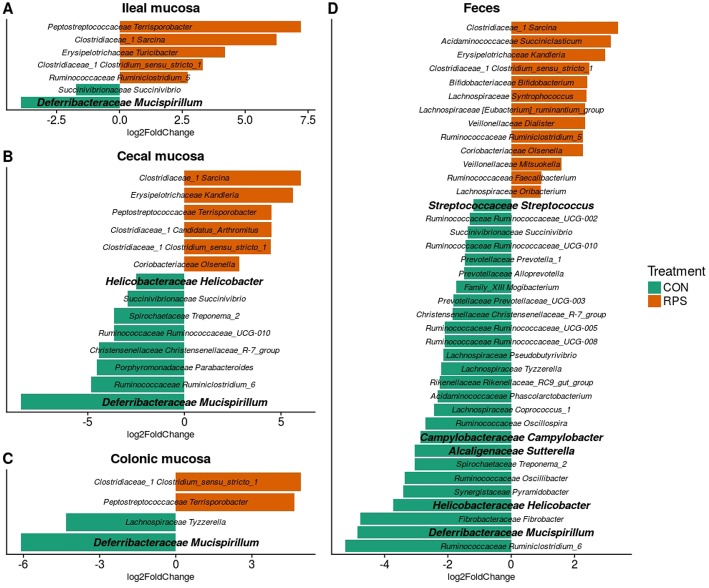
Differentially abundant (*p* < 0.05) genera based on 16S rRNA gene sequences from the ileal mucosa **(A)**, cecal mucosa **(B)**, colonic mucosa **(C)**, and feces **(D)** as determined by DeSeq2. OTUs clustered at 97% similarity were combined by taxonomic classification at the genus level. The results shown are log_2_ fold change between the CON (control; green) and RPS (resistant potato starch; orange)-fed groups; note that the x-axis scale is different for each panel. Positive log-fold changes indicate that a genus is enriched in the RPS group, while negative log-fold changes indicate that a genus is enriched in the CON group. The SILVA classification for each genus is labeled on the figure using both the family and genus name. Genera shown in bold are those that harbor species with the capacity for respiration.

**Figure 4:**
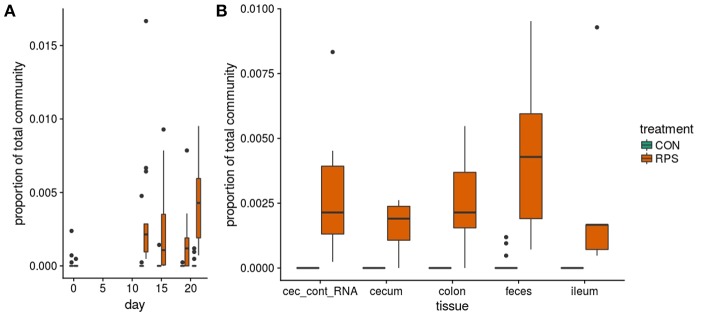
The abundance of OTU0087 over time **(A)** and in intestinal tissues at day 21 **(B)** in CON (green) or RPS (orange)-fed pigs. 16S rRNA gene sequences from this OTU most closely matched those from *Clostridium chartatabidum*, a member of the genus *Clostriduim sensu stricto 1*. All comparisons between treatment groups are significant by Wilcoxon tests *p* < 0.05.

At day 21 post-weaning, many bacterial genera were significantly differentially abundant at several intestinal sites between the treatments (Figure 3). Several genera were consistently associated with each respective treatment in most sampling locations. Pigs fed RPS had significantly increased levels of *Terrisporobacter, Sarcina*, and *Clostridium sensu stricto 1* compared to CON-fed pigs. One OTU from the genus *Clostridium sensu stricto* was strongly associated with the RPS treatment group; OTU0087 was significantly enriched in RPS fed animals at all-time points and tissues (Figure 4). Sequences within this OTU most closely match *Clostridium chartatabidum*. CON-fed pigs had a significant enrichment of *Mucispirillum*, as well as occasional enrichment of various *Proteobacteria* genera such as *Helicobacter, Sutterella*, and *Campylobacter* in different tissues.

Similar to the 16S rRNA gene sequence data, many *but* gene-based OTUs were differentially represented between the two groups (Figure S3). RPS intake was consistently associated with a greater abundance of *but* OTUs whose sequences most closely matched to those from *Anaerostipes hadrus*, as well as a *but* OTU whose sequences most closely matched an organism detected in metagenomes from human feces (*but* OTU0067). Importantly we also measured *but* gene transcripts in the cecal contents and found that these same OTUs were significantly enriched in the transcript data as well. Collectively, these results suggest that particular butyrate-producing bacteria were enriched by RPS intake, and their metabolic activities were likely responsible for increased butyrate concentrations detected in the intestinal samples from these animals.

### Bacterial Metabolites Differed Between the Treatment Groups

**Figure 5:**
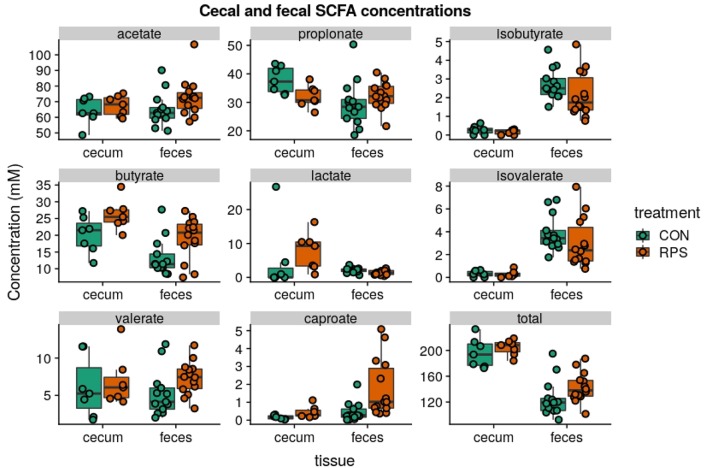
SCFA concentrations (mM) from cecal contents and feces at day 21 post-weaning. Total SCFAs are the sum of the following SCFAs: formate, acetate, propionate, isobutyrate, butyrate, lactate, isovalerate, valerate, caproate, oxalate, phenylacetate, succinate, and fumarate. Group differences were tested with Wilcoxon tests, notable differences were as follows: butyrate: cecum *p* = 0.05 & feces *p* = 0.05, caproate: cecum *p* = 0.07 & feces *p* = 0.001, propionate: cecum *p* = 0.05, lactate: cecum *p* = 0.02 and feces *p* = 0.09, total: feces *p* = 0.03.

Short-chain fatty acid (SCFA) concentrations in cecal and fecal samples collected at 21-days post-weaning were evaluated to investigate whether the changes in bacterial communities had an impact on microbial metabolic output. Aligning with the changes in microbial community structure, the CON and RPS-fed pigs had differing SCFA profiles (Figure 5). Pigs fed RPS had higher amounts of butyrate in both the cecum and feces (*p* = 0.05 & *p* = 0.05), higher levels of caproate in both the cecum and feces (*p* = 0.07 & *p* = 0.001), lower levels of propionate in the cecum (*p* = 0.05), lower levels of lactate in the feces (*p* = 0.02), higher amounts of lactate in the cecum (*p* = 0.09). In the cecum, one animal in the CON group had very high levels of lactate, strongly diverging from the levels found in the rest of the CON group, and removing this animal from this particular comparison resulted in a significant difference (*p* = 0.02) for cecal lactate levels. Total SCFA concentrations in cecal contents were not different between the treatments, but total fecal SCFAs were significantly increased in the RPS group (*p* = 0.03). Collectively, dietary RPS modulated bacterial community structure in the distal gut, and community differences were associated with different SCFA levels.

### Differential Host Response With Dietary RPS

To investigate the impact of dietary RPS on immune status, and to correlate detected immune changes with bacterial community alterations, T cell populations in the cecum, ileocecal lymph node, and peripheral blood were phenotyped by flow cytometry. From this analysis we did not detect a significant difference in the abundance of CD3^+^ cells in any sample type between treatment groups, and the lack of change to the number of CD3^+^ cecal cells with dietary RPS was supported by IHC results (Figure S4). In the cecum, <1% of the CD3^+^ cells labeled with MAC320 monoclonal antibody, a marker of peripheral γδ T cells (33, 34) (Figure S2), suggesting that the majority of T cells in the cecum were αβ T cells.

**Figure 6:**
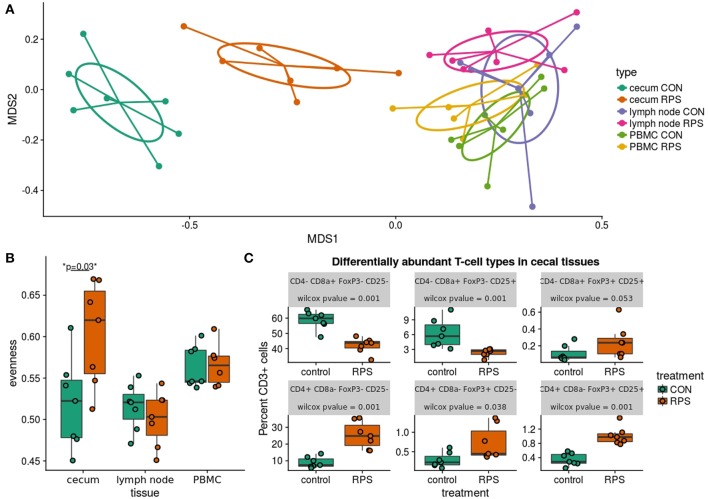
Differences in T cell populations between the RPS and CON animals at 21 days post-weaning. **(A)** NMDS ordination of Bray-Curtis dissimilarities of T cell communities in various tissues. Each point represents the total T cell community for one pig in a certain tissue, and distances between points represent the similarities between communities. **(B)** The evenness (Pielou's J) of the T cell communities in various tissues. Low evenness indicates that a community is dominated by a few abundant members. **(C)** Boxplots of significantly differentially abundant T cell types from the cecal T cell community.

Subsequent analysis of cecal T cell populations revealed distinct changes associated with dietary treatment. A panel of antibodies against CD3, CD4, CD8α, CD25, and FoxP3 were used to simultaneously identify 16 distinct T cell populations (Table S6). The relative abundance of each cell type was reported as a percent of the total CD3^+^ cells, generating a community data matrix for ecological analyses. No significant differences between groups were observed in T cell communities in the ileocecal lymph node or peripheral blood; however, a significant difference in overall cecal T cell community structure was detected between treatment groups (Figure 6A) (PERMANOVA *p* = 0.001, *F* = 12.06). Additionally, the evenness of the cecal T cell communities in CON animals was significantly reduced, meaning these communities tended to be dominated by a few cell types (Figure 6B, *p* = 0.02). Several types of cecal T cells were differentially abundant between the treatments (Figure 6C). More specifically, we observed an increase in several CD8α^+^ populations and a relative decrease in FoxP3^+^ cells in CON animals. These data suggest that animals fed RPS had increased abundances of T regulatory cell types associated with immune tolerance; however, these changes were limited to the cecal mucosa.

**Figure 7:**
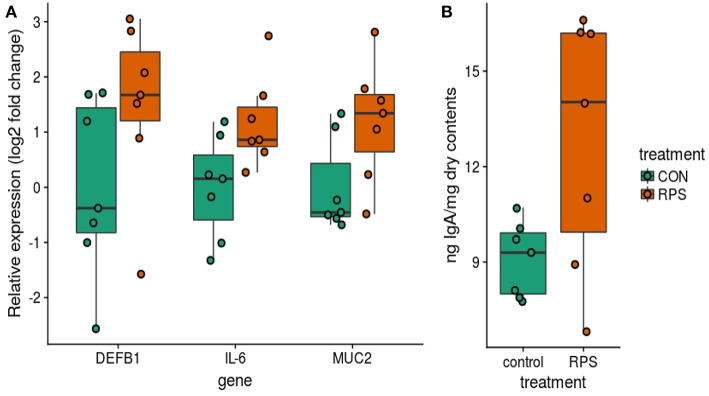
Differences in host traits associated with mucosal barrier function between CON and RPS-fed animals. **(A)** mRNA expression of barrier function-associated genes from the cecal mucosa. **(B)** Amount of IgA in cecal contents. Wilcoxon *p-*values: DEFB1 *p* = 0.16, IL-6 *p* = 0.04, MUC2 *p* = 0.04, IgA *p* = 0.1. DEFB1 = beta defensin 1, IL-6 = Interleukin 6 gene, MUC2 = mucin 2 gene. IgA = Immunoglobulin A.

In addition to differences in the cecal T cell populations, significant differences in the expression of genes important for barrier function were detected in the cecal mucosa. Significantly greater expression of MUC2 and IL6 was observed (*p* = 0.04 and *p* = 0.04), as well as a trend toward increased expression of DEF1B (Wilcox *p* = 0.16, T. test *p* = 0.10) in the RPS-fed group compared to the CON group (Figure 7A). Intestinal IgA is another important host-produced factor that enhances intestinal barrier function; therefore, cecal luminal contents were assayed for total IgA concentration. RPS-fed pigs trended toward increased cecal luminal IgA at necropsy (Wilcox *p* = 0.09, T. test *p* = 0.05) (Figure 7B). No increase in the number of IgA^+^ cells in cecal tissues was detected (Figure S4), suggesting increased secretion of IgA from plasma cells in the cecum.

### Bacterial Members and Metabolites Correlated With Cecal Immune Status

**Figure 8:**
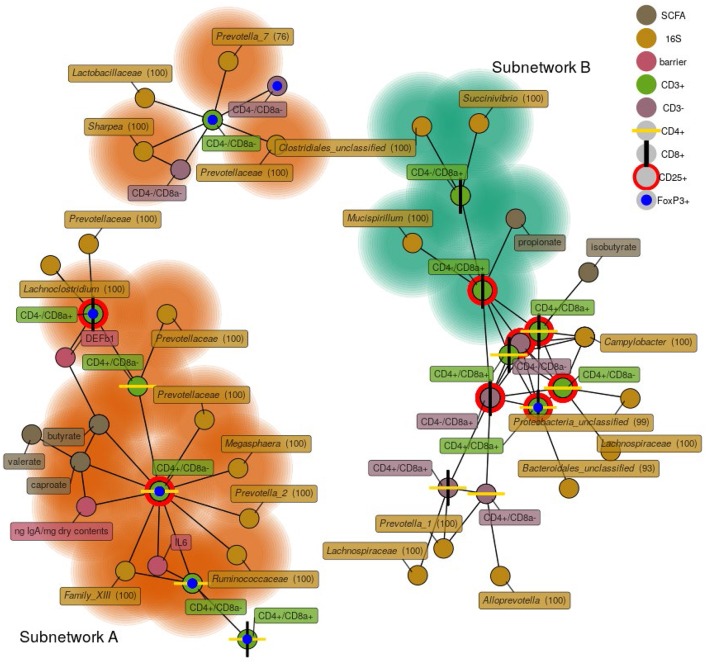
A network depicting correlations among cecal immune cells, 16S rRNA gene OTUs, cecal SCFA concentrations, cecal host mRNA expression, and cecal IgA concentrations. Only positive correlations are shown with a Spearman coefficient > 0.6 and a *p* < 0.05. Bacterial nodes are OTUs labeled with their taxonomic classification according to the SILVA database. Labels are genus names except when no classification at the genus level was possible, in which case family names are shown. Nodes shaded with green are features that are enriched in the CON group, nodes shaded in orange are those enriched in the RPS group.

To investigate relationships among bacterial membership, bacterial function, and host immune status in the cecum, a correlation network was constructed using the relative abundance of cecal cells [both CD3^+^ and CD3^−^ cell types were used in this analysis (Table S7)], cecal 16S rRNA gene OTU abundance, cecal SCFA concentrations, cecal tissue gene expression, and luminal IgA concentrations (Figure 8). The results showed one discrete subnetwork associated with each respective treatment group. The subnetwork associated with pigs fed RPS (labeled subnetwork-A) was composed of features associated with immune tolerance, mucosal barrier function, and anaerobic microbial fermentation. Classic T-regulatory (CD3^+^CD4^+^CD8α^−^CD25^+^FoxP3^+^) cells formed a central node in the RPS subnetwork along with several other CD3^+^FoxP3^+^ cell-types. Concentrations of the SCFAs butyrate, caproate, and valerate correlated with these regulatory T cells. Bacterial OTUs in the RPS subnetwork belonged to groups known for anaerobic fermentation, and several OTUs corresponded to known butyrate-producing bacterial groups, such as the genus *Megasphaera* and the family *Ruminococcaceae*. In addition, subnetwork-A contained nodes associated with an enhanced mucosal barrier: DEF1B, and IL-6 expression, and high luminal IgA concentrations.

The subnetwork associated with the CON diet (labeled subnetwork-B) was composed of markedly different features defined by immune activation, cytotoxic T cells, and bacteria capable of respiration. Not all of the features of subnetwork-B were significantly enriched in the CON pigs. Only cytotoxic T cells (both CD25^+^ and CD25^−^), propionate, a *Succinivibrio* OTU, and a *Mucispirillum* OTU were enriched in the CON diet. The core of subnetwork-B was composed of highly interconnected nodes, mainly cells expressing CD25 and a *Campylobacter* OTU, suggesting T cell activation and conditions conducive to microbial respiration. Several of the OTUs in subnetwork-B belong to the *Proteobacteria* phylum, members of which are known to use respiratory metabolisms (35). These results suggest that dietary RPS enhanced bacterial production of SCFAs that benefited host health by promoting epithelial barrier function, increased immune tolerance, and a reduced niche for microbial respiration relative to CON-fed pigs.

## Discussion

RPS is an accessible metabolic substrate for intestinal microbes and can lead to increased production of SCFAs, particularly butyrate, which have beneficial effects on the gastrointestinal system (36). Our results showed increased concentrations of cecal butyrate and lactate in RPS-fed animals compared to the CON animals. In the distal gut, lactate is converted to butyrate (37), and the lactate observed in the cecum was likely converted to butyrate during colonic passage as fecal lactate concentrations were very low. Supporting this idea, we observed an enrichment of both lactate producing bacterial genera as well as butyrate–producing bacteria known to consume lactate, including *Anaerostipes hadrus* and *Megasphaera elsdenii*, in RPS-fed animals. Butyrate is well-established as a bacterial metabolite of central importance for intestinal homeostasis that supports many aspects of gut health, including reducing the mucosal niche for bacterial respiration (13), increasing barrier function (6, 38), and encouraging an appropriate immune response, skewing toward T cell tolerance of symbiotic microbes (39, 40).

The effects of prebiotics such as RPS are mediated through the microbiota, consequently the impact can vary greatly depending on the initial composition of intestinal bacterial communities. The large inter-individual variation present in the intestinal microbiota of mammals is well-documented, swine included (41). Several studies investigating dietary resistant starch indicate prebiotic responders and non-responders can be grouped by the presence of certain microbial members (16, 17, 42). Therefore, to optimize the effect of dietary prebiotics it may be necessary to ensure that the appropriate microbial food webs are present in the host. One study suggests that co-administration of resistant starch and a probiotic strain of *Bifidobacteria* spp. produced more desirable health outcomes than resistant starch alone (43). Our work identifies swine gut microbiota members that could be co-administered alongside RPS to potentially enhance its beneficial effects.

RPS-enriched bacteria are mainly organisms known to use fermentative metabolisms. Well-known fermenters such as *Bifidobacteria* spp. and *Faecalibacterium* spp. were enriched in the RPS-fed animals and are associated with intestinal health in humans (44), and other studies have shown increased abundance of these genera in pigs fed resistant starch (3, 45). Some genera enriched in the RPS-fed animals are not well-characterized for their health benefits. For example, members of the genus *Clostridium sensu stricto 1* are not generally associated with positive health outcomes; however, our data suggest that members of this genus may be important for resistant starch degradation in the distal gut. In particular 16S rRNA gene OTU0087 was greatly enriched in the RPS group and most closely matched *Clostridium chartatabidum*. Bacteria similar to this organism have been isolated from chemostats fed with resistant starch and inoculated with human feces (46), additionally a recent study suggests that bacteria falling within this taxonomic group were important for a response to RPS in a human feed trial (17). These data suggest that certain bacterial groups likely occupy similar niches in different host species.

Many of the differences between the treatment groups were likely a result of feedback interactions between intestinal bacterial metabolites and host tissues. Butyrate (and other SCFAs) produced by gut bacteria is oxidized by host tissues, thereby limiting the amount of oxygen available at the mucosa (13). This establishes a mucosal environment favoring microbial fermentation over respiration, and therefore more SCFA production. Increased mucosal tolerance additionally limits the release of immune-associated electron acceptors (12). In total these effects limit the niche for microbial respiration, and the types of bacteria enriched in the RPS-fed animals compared to the CON-fed animals are consistent with this model.

Increases in beneficial bacterial populations and metabolites in the RPS-fed animals were associated with some indications of positive impacts on the mucosal barrier (a component of intestinal innate immunity) as well as increased numbers of immune regulatory cells in the cecum. Some markers of mucosal barrier function, in particular MUC2, IL-6, and a trend toward increased DEFB1, were enhanced in the cecal mucosa of pigs fed RPS. IL-6 was recently described as critical for maintaining the mucosal barrier (47) and also important for strong IgA responses (48). Alterations in innate immune gene expression at the mucosal level may be the result of an altered microbiota and subsequent changes in abundance of different microbe-associated molecular patterns (MAMPS). Recognition of MAMPS via recognition receptors at the intestinal epithelial can alter expression of antimicrobial peptides (such as DEFB1) and mucus (MUC2) (49). T cell subtypes, in particular T-regulatory cells (T-regs), and indicators of intestinal health and mucosal barrier function significantly correlated with microbial butyrate and caproate production, as well as several anaerobic *Clostridial* OTUs in the cecal mucosa of the RPS-fed animals. Butyrate is known to induce *de novo* generation and expansion of peripheral T-regs, reviewed in Zeng et al. which are critical for intestinal homeostasis and gut barrier function (39). T-regs moderate immune responses to commensal microbes, reducing intestinal inflammation and subsequent mucosal availability of immune-derived electron acceptors, thereby limiting microbial respiration (13). T-regs can promote mucosal IgA responses, helping the host exert control over its microbial partners (39, 50, 51), and cecal luminal IgA levels tended to be increased in RPS-fed pigs and correlated with the abundance CD4^+^ T-regulatory cells. CD8α^+^ T-regulatory cells (CD4^−^ CD8α^+^ CD25^+^ FoxP3^+^) are less well-studied than CD4^+^ T-regs, but recent work indicates they are an important regulatory cell type in humans and mice (52), and we detected these cells in the cecum of pigs. Though their direct role in intestinal health is unclear, the network analysis suggests that animals with greater relative abundance of CD8^+^ T-regs also had higher expression of DEFB1.

Though they were healthy, the CON group exhibited signs of reduced immune tolerance and greater abundances of potentially invasive bacteria in their mucosal tissues relative to the RPS group. Specifically, CON-fed animals had more cytotoxic T cells (CD8α^+^ cell types) compared to RPS-fed pigs, although they were unlikely active given the lack of pathological changes in the cecum. Similarly, bacteria enriched in the CON animals have previously been associated with intestinal inflammation, dysbiosis in humans and mice (53), and utilize respiration as their preferred metabolism. In particular, members of the genus *Mucispirillum* have been shown to thrive in inflamed, oxygenated mucosal environments (54). Our results showing correlations between the abundance of *Mucispirillum* and cytotoxic T cells suggest that immune cell activity may play a role in expanding the niche for this organism in the CON pigs. Similarly, we detected enrichment of *Helicobacter* 16S rRNA in the cecum and feces from CON-fed animals. Bacteria from this genus can be facultative intracellular pathogens (55), and it has been proposed that non-*H. pylori Helicobacter* species may be a cause of irritable bowel disease (IBD) in humans (56). These observations suggest that though the CON pigs lacked distinct pathology, the mucosae of these animals were more amenable to colonization by potentially pathogenic organisms that utilize respiratory metabolisms, relative to the RPS pigs.

Local intestinal inflammatory responses have previously been shown to occur in swine early in weaning (57), and though no overt inflammation was observed in these tissues in the current study, we detected immune cell types associated with recent immune activity in both treatment groups. In the cecal tissue network analysis, many nodes residing in subnetwork B were equally represented in both the CON and RPS groups. For example, double-positive (CD4^+^CD8α^+^) CD25^+^ T cells were central members of subnetwork B and were not differentially abundant between treatments; these cells have been shown to be common in swine and represent activated, memory effector cells (58). Interestingly, a *Campylobacter* OTU, was correlated with the abundance of these cells and several other CD25^+^ cell types. This observation suggests that certain bacterial groups, particularly *Proteobacteria*, may benefit from the local environmental changes associated with recent immune activity, such as the release of reactive nitrogen or oxygen species. While the memory cells may not be the source of reactive nitrogen or oxygen species, their presence indicates prior perturbation of the mucosal site. The idea that *Proteobacteria* thrive using products of the immune response is well-established for *Salmonella* in mice (11, 12) and our data suggest that this model warrants further investigation for other bacterial species and hosts.

## Conclusions

This study demonstrated that dietary intake of RPS had beneficial impacts on the intestinal health status of weaning pigs, including increased markers of mucosal barrier function, immune tolerance, and increased abundances of potentially beneficial bacterial populations. Our study suggested that RPS was initially fermented to lactate by various lactic acid bacteria and then into butyrate and other SCFAs by secondary fermenters such as *Anaerostipes hadrus*. Members of these bacterial groups that could potentially be co-administered with RPS to enhance its effects. Additionally, this work revealed specific correlated changes between the commensal microbiota and the mucosal immune system that can be used to inform future strategies to modulate the microbiota to support health. These data provide valuable insights into the host-microbe interactions in the intestinal mucosa of swine, furthering our knowledge of the mammalian hindgut ecosystem. Finally, pigs are recognized as a relevant translational model for human health, and research to enhance intestinal health in pigs provides insights for enhancing human health as well.

## Ethics Statement

This study was carried out in accordance with the recommendations of USDA-ARS-National Animal Disease Center Institutional Animal Care and Use Committee, and was approved by the USDA-ARS-NADC IACUC.

## Author Contributions

JT, CL, and HA conceived of and planned the study. NG formulated and prepared the diets, and provided guidance on diet phases. CL and CB collected the gene expression, flow cytometry, IgA, and immunohistochemistry data. JT collected microbial community data and SCFA data. JT performed the data analysis. JT, CB, HA, and CL wrote and revised the manuscript.

### Conflict of Interest Statement

The authors declare that the research was conducted in the absence of any commercial or financial relationships that could be construed as a potential conflict of interest.
