# Supplementary material for: Dietary Resistant Potato Starch Alters Intestinal Microbial Communities and Their Metabolites, and Markers of Immune Regulation and Barrier Function in Swine
Source: Front Immunol. 2019 Jun 19;10:1381. doi: 10.3389/fimmu.2019.01381 (PMC6593117; doi:10.3389/fimmu.2019.01381)
Supplement: Supplementary file 1 [file Data_Sheet_1.pdf]

Table S1: Composition of diets. All diets were formulated at Iowa State University.

	CON Phase I Diet	RPS Phase 1 Diet	CON Phase 2 Diet	RPS Phase 2 Diet
Corn, yellow dent	49.00%	44.00%	61.06%	56.06%
Soybean meal (46.5% CP)	28.20%	28.20%	27.00%	27.00%
Casein	2.70%	2.70%		-
Lactose	10.00%	10.00%	2.50%	2.50%
Fish meal (menhaden)	4.50%	4.50%	4.66%	4.66%
Soybean oil	2.00%	2.00%	1.65%	1.65%
L-lysine HCl	0.35%	0.35%	0.38%	0.38%
DL-methionine	0.15%	0.15%	0.12%	0.12%
L-threonine	0.13%	0.13%	0.12%	0.12%
L-tryptophan	0.02%	0.02%	0.01%	0.01%
L-valine	0.11%	0.11%	-	-
Monocalcium phosphate (21%)	1.15%	1.15%	0.96%	0.96%
Limestone	0.80%	0.80%	0.74%	0.74%
Salt	0.50%	0.50%	0.40%	0.40%
NSNG Nursery Vitamin Premix	0.25%	0.25%	0.25%	0.25%
NSNG Trace Mineral Mix[a]	0.15%	0.15%	0.15%	0.15%
MSP[RS] Resistant Starch	-	5.00%	-	5.00%

[a]: National Swine Nutrition Guide

Table S2: Basic information about the animals used in this study

Pig Number	Sow	Sex	Diet	Birth Weight	Final Weight
67	1211	F	CON	4	11.1
68	1214	M	CON	5.6	12
69	1213	M	CON	5	7.4
70	1210	F	CON	5.9	12
71	1209	F	CON	7	11.5
72	1207	F	CON	8.5	15.8
73	1211	M	CON	3.7	7.9
74	1211	F	RPS	3.2	8.6
75	1214	M	RPS	5.4	11.8
76	1213	F	RPS	4.4	7.8
77	1210	M	RPS	6.1	9.7
78	1209	F	RPS	7	13.6
79	1214	F	RPS	5.6	11.8
80	1211	F	RPS	4.5	11.7
81	1212	F	CON	4.9	Not Necropsied
82	1215	M	CON	6	Not Necropsied
83	1210	F	CON	6.3	Not Necropsied
84	1213	F	CON	5.5	Not Necropsied
85	1211	M	CON	4.7	Not Necropsied
86	1214	F	CON	4.8	Not Necropsied
87	1208	F	CON	6.4	Not Necropsied
90	1215	M	RPS	7.3	Not Necropsied
91	1210	M	RPS	6.7	Not Necropsied
92	1213	F	RPS	4.6	Not Necropsied
93	1211	M	RPS	4.5	Not Necropsied
94	1214	M	RPS	5.3	Not Necropsied
95	1208	M	RPS	7.2	Not Necropsied
96	1207	M	RPS	7.8	Not Necropsied

Table S3: TaqMan® Gene Expression Targets for qRT-PCR*.*

Symbol	Gene Name	Accession	Assay ID	Function
DEFB1	defensin β1	NM_213838	Ss03381769_u1	antimicrobial peptide
FFAR2	free fatty acid receptor 2	NM_001278758	Ss03374174_s1	SCFA receptor
IL-10	interleukin-10	NM_214041	Ss03382372_u1	cytokine signaling
IL-17	interleukin-17A	NM_001005729	Ss03391803_m1	cytokine signaling
IL-22	interleukin-22	AY937228	Ss03373919_m1	cytokine signaling
IL-1β	interleukin-1β	NM_214055	Ss03393804_m1	cytokine signaling
IL-6	interleukin-6	NM_214399	Ss03384604_u1	cytokine signaling
MUC2	small intestinal mucin	EU143549	Ss03377386_u1	mucus layer defense
PR39	PR-39	NM_214450	Ss03385004_u1	antimicrobial peptide
TGFβ	transforming growth factor β	NM_214015	Ss03382325_u1	cytokine signaling
ACTβ	β -actin	AK237086	Ss03376081_u1	reference gene

All primers were labeled with FAM dye and specific for s*us scrofa* (Thermo Fisher Scientific).

Figure S1: Representative flow plot used for determining frequency of T cell populations in the cecum. 1A) Live cells gating (negative for stain), 1B) $CD3^{+}$ gating 1C) CD4 and Cd8α gating of live $CD3^{+}$ cells and 1D) representative gating for CD25 and FoxP3 staining of indicated T cell populations - the blue dots are representative of $CD4^{+}$/ $CD8\alpha^{-}$ population and the red dots are representative of $CD4^{+}$/ $CD8\alpha^{+}$ population. 1E) gd TCR staining

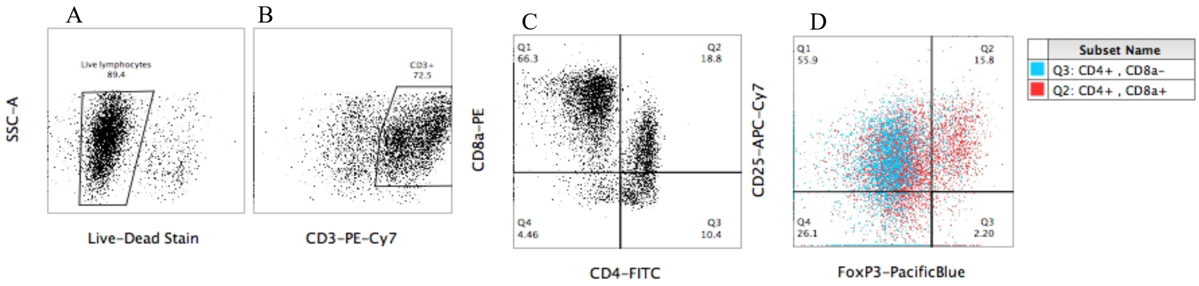

Figure S2: Representative flow plots depicting γδ-TCR staining in A: PBMCs, B: Ileocecal lymphnode, C: cecal mucosa. Less than 1% of CD8+ events in the cecal mucosa were also γδ +

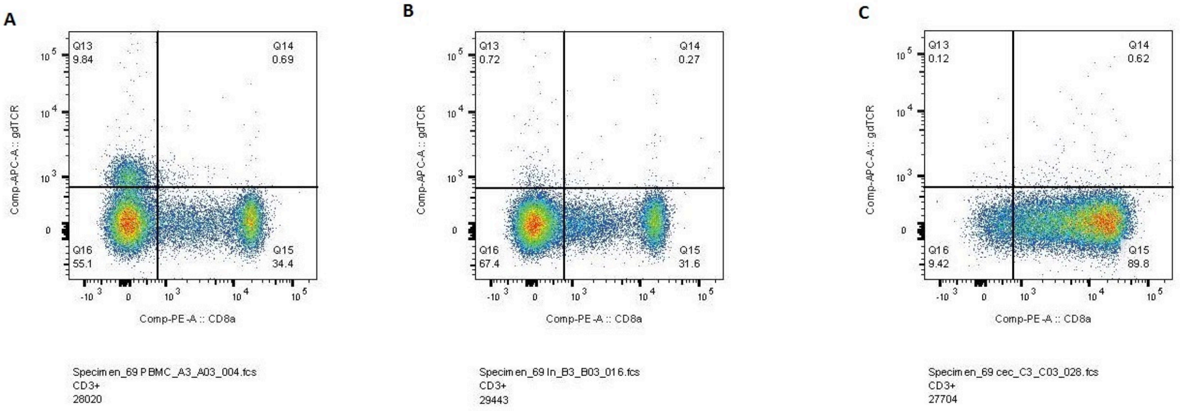

Table S4: PERMANOVA results showing treatment group differences in community structure across time and tissues. Both 16S and *but* based communities. Calculated with vegan's Adonis() function.

Comparison	F.Model	R2	p.value	gene	day	tissue
feces_0_control vs feces_0_RPS	0.597851	0.022477	0.9073	16S	0	feces
feces_12_control vs feces_12_RPS	0.974797	0.037529	0.4307	16S	12	feces
feces_15_control vs feces_15_RPS	1.94773	0.072278	0.0159	16S	15	feces
feces_19_control vs feces_19_RPS	2.607228	0.105954	0.0017	16S	19	feces
feces_21_control vs feces_21_RPS	5.257901	0.179709	1.00E-04	16S	21	feces
cec_cont_RNA_21_control vs cec_cont_RNA_21_RPS	1.860549	0.134233	0.0566	16S	21	cec_cont_RNA
cecum_21_control vs cecum_21_RPS	2.64312	0.180503	0.0085	16S	21	cecum
colon_21_control vs colon_21_RPS	1.441706	0.107256	0.1439	16S	21	colon
ileum_21_control vs ileum_21_RPS	2.67778	0.308579	0.0367	16S	21	ileum
feces_0_control vs feces_0_RPS	0.794	0.029633	0.5793	*but*	0	feces
feces_12_control vs feces_12_RPS	0.746653	0.027916	0.7542	*but*	12	feces
feces_15_control vs feces_15_RPS	1.522014	0.057387	0.0925	*but*	15	feces
feces_19_control vs feces_19_RPS	3.582779	0.151924	1.00E-04	*but*	19	feces
feces_21_control vs feces_21_RPS	3.880003	0.129853	3.00E-04	*but*	21	feces
cec_cont_RNA_21_control vs cec_cont_RNA_21_RPS	1.694801	0.133504	0.054	*but*	21	cec_cont_RNA
cecum_21_control vs cecum_21_RPS	1.859664	0.134178	0.0483	*but*	21	cecum
colon_21_control vs colon_21_RPS	1.36554	0.102169	0.2063	*but*	21	colon
ileum_21_control vs ileum_21_RPS	2.333303	0.162789	0.0112	*but*	21	ileum

Table S5: Community dispersions across time and tissues within and between groups for both 16S and *but* communities. Calculated with a permutation test of vegan's betadisper() function.

Comparison	16S p value	but p value
cec_cont_RNA_21_control vs cec_cont_RNA_21_RPS	0.886	0.028
cecum_21_control vs cecum_21_RPS	0.007	0.012
colon_21_control vs colon_21_RPS	0.129	0.078
feces_0_control vs feces_0_RPS	0.942	0.714
feces_0_control vs feces_12_control	0.299	0.758
feces_0_control vs feces_12_RPS	0.009	0.638
feces_0_control vs feces_15_control	0.001	0.744
feces_0_control vs feces_15_RPS	0.001	0.443
feces_0_control vs feces_19_control	0.003	0.064
feces_0_control vs feces_19_RPS	0.002	0.538
feces_0_control vs feces_21_control	0.001	0.223
feces_0_control vs feces_21_RPS	0.001	0.033
feces_0_RPS vs feces_12_control	0.409	0.934
feces_0_RPS vs feces_12_RPS	0.042	0.962
feces_0_RPS vs feces_15_control	0.001	0.502
feces_0_RPS vs feces_15_RPS	0.001	0.25
feces_0_RPS vs feces_19_control	0.003	0.044
feces_0_RPS vs feces_19_RPS	0.009	0.353
feces_0_RPS vs feces_21_control	0.004	0.126
feces_0_RPS vs feces_21_RPS	0.001	0.013
feces_12_control vs feces_12_RPS	0.351	0.873
feces_12_control vs feces_15_control	0.015	0.464
feces_12_control vs feces_15_RPS	0.032	0.22
feces_12_control vs feces_19_control	0.081	0.02
feces_12_control vs feces_19_RPS	0.125	0.258
feces_12_control vs feces_21_control	0.183	0.065
feces_12_control vs feces_21_RPS	0.001	0.003
feces_12_RPS vs feces_15_control	0.056	0.352
feces_12_RPS vs feces_15_RPS	0.189	0.139
feces_12_RPS vs feces_19_control	0.32	0.007
feces_12_RPS vs feces_19_RPS	0.429	0.181
feces_12_RPS vs feces_21_control	0.577	0.04
feces_12_RPS vs feces_21_RPS	0.001	0.004
feces_15_control vs feces_15_RPS	0.272	0.686
feces_15_control vs feces_19_control	0.341	0.108
feces_15_control vs feces_19_RPS	0.264	0.856
feces_15_control vs feces_21_control	0.175	0.363
feces_15_control vs feces_21_RPS	0.054	0.072
feces_15_RPS vs feces_19_control	0.932	0.135
feces_15_RPS vs feces_19_RPS	0.748	0.775
feces_15_RPS vs feces_21_control	0.543	0.529
feces_15_RPS vs feces_21_RPS	0.001	0.075
feces_19_control vs feces_19_RPS	0.839	0.055
feces_19_control vs feces_21_control	0.645	0.283
feces_19_control vs feces_21_RPS	0.005	0.714
feces_19_RPS vs feces_21_control	0.803	0.281
feces_19_RPS vs feces_21_RPS	0.001	0.041
feces_21_control vs feces_21_RPS	0.003	0.175
ileum_21_control vs ileum_21_RPS	0.799	0.569

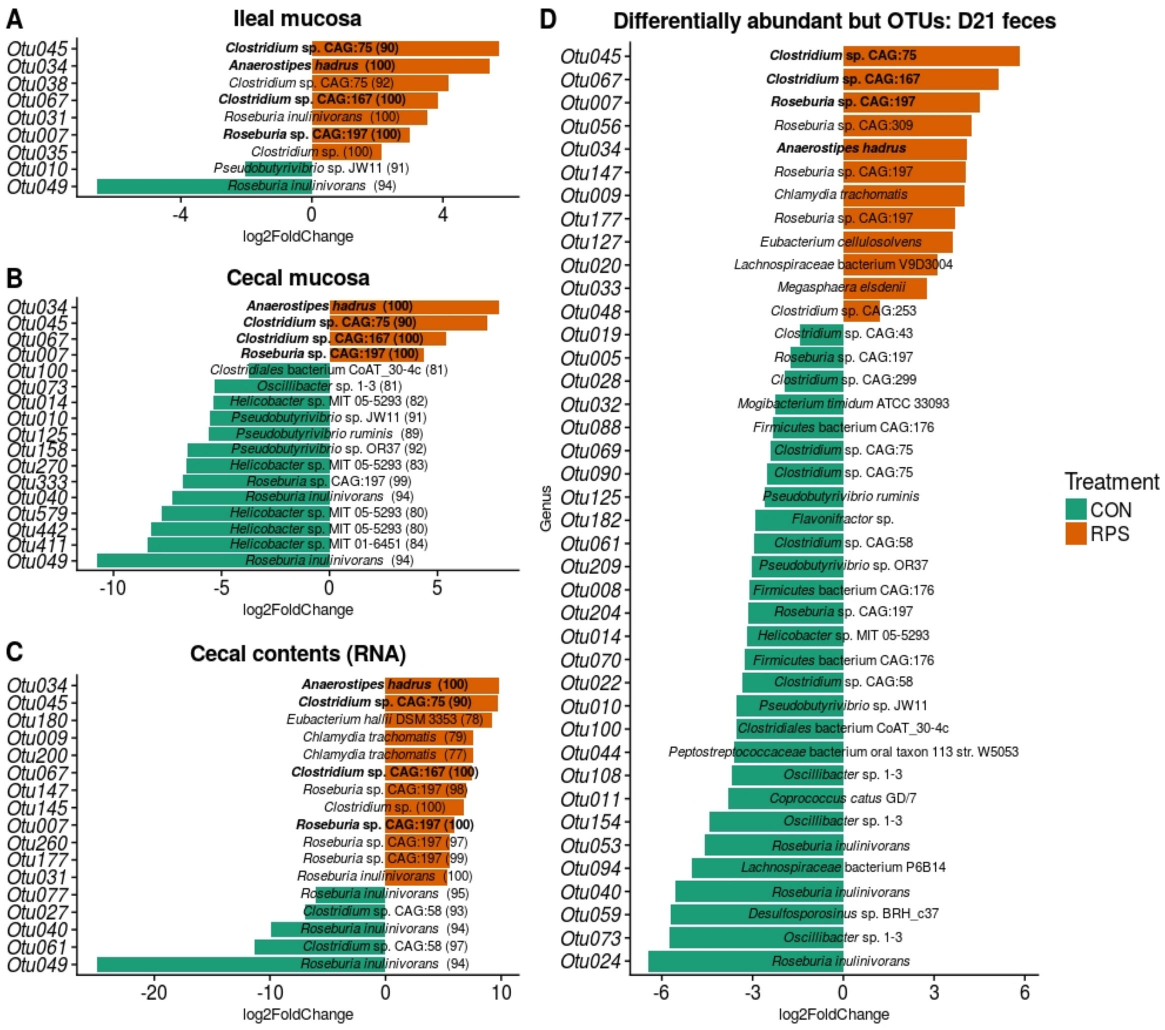

Figure S3: Significantly ($p<0.05$) differentially abundant but gene OTUs from the ileal mucosa (A), cecal mucosa (B), colonic mucosa (C), and feces (D) as determined by DeSeq2. The results shown are log2 fold change between the CON (control) and RPS (resistant potato starch)-fed groups; note that the x-axis scale is slightly different for each panel. Positive log fold changes indicate that OTU is enriched in the RPS group while negative log fold changes indicate that OTU is enriched in the CON group. OTUs were clustered at 97% similarity and are labeled with the species name for their closest BLAST hit followed by the percent identity for that hit. OTUs labeled in bold are those which were consistently enriched in one treatment group or the other.

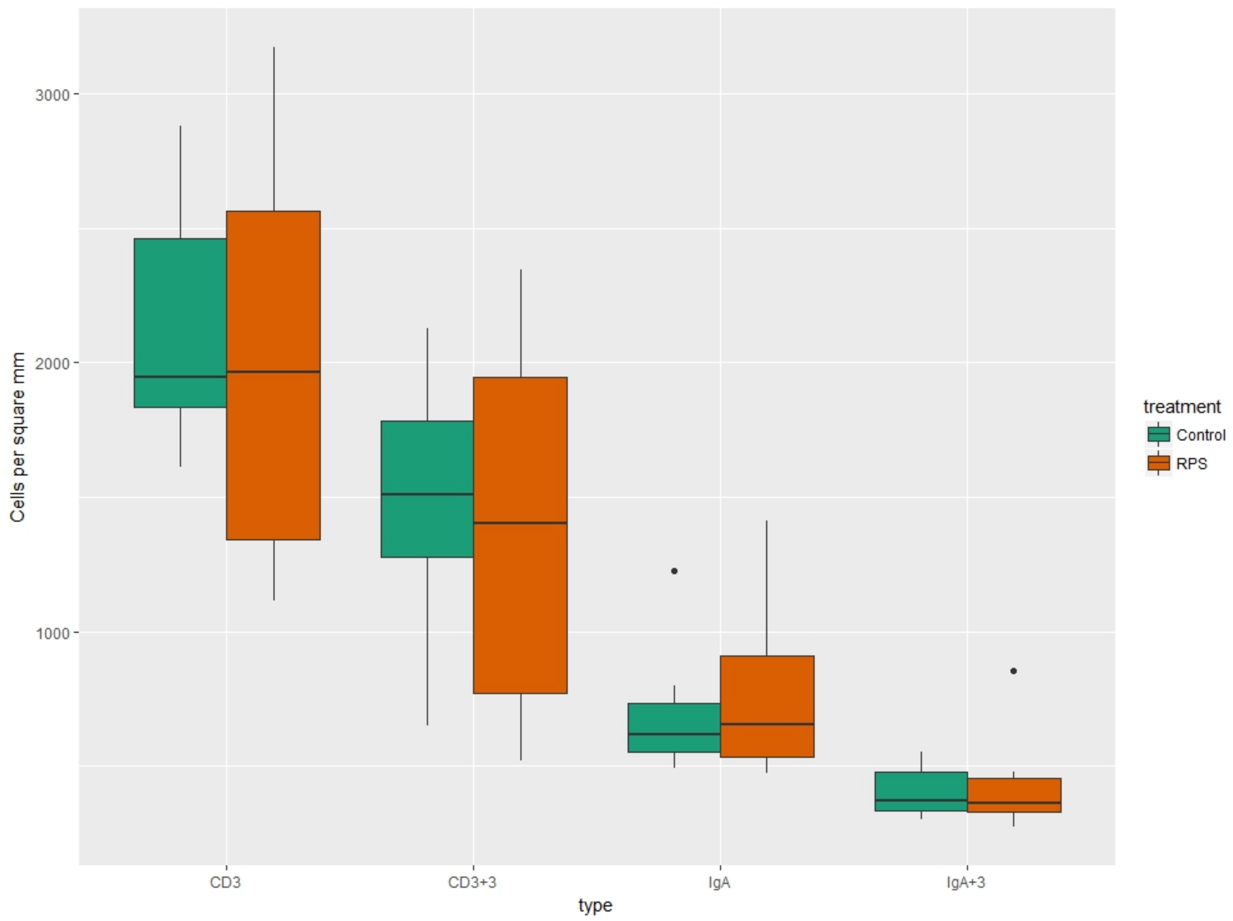

Figure S4: CD3+ and IgA+ cells as determined by IHC. No group differences were apparent for either cell type. The “+3” cell types refer to cells that met a high staining intensity threshold in the automated Aperio software

Table S6: Percent abundance of each CD3+ cell type detected in each tissue by our flow panel. These data are an aggregate of all animals (combining the treatment groups)

	% abundance in each Tissue		
**Cell type**	**Cecum**	**lymph-node**	**PBMC**
CD3+CD4-CD8a-FoxP3-CD25-	7.7	16.9	23.1
CD3+CD4-CD8a-FoxP3-CD25+	0.6	0.7	1.5
CD3+CD4-CD8a-FoxP3+CD25-	0.3	0.1	0.1
CD3+CD4-CD8a-FoxP3+CD25+	0.1	0.2	0.2
CD3+CD4-CD8a+FoxP3-CD25-	50.4	25.1	25.2
CD3+CD4-CD8a+FoxP3-CD25+	4.3	0.2	0.5
CD3+CD4-CD8a+FoxP3+CD25-	0.1	0.1	0.2
CD3+CD4-CD8a+FoxP3+CD25+	0.2	0.4	0.4
CD3+CD4+CD8a-FoxP3-CD25-	17.2	46.9	35.9
CD3+CD4+CD8a-FoxP3-CD25+	1.4	0.5	0.4
CD3+CD4+CD8a-FoxP3+CD25-	0.5	0.3	0.3
CD3+CD4+CD8a-FoxP3+CD25+	0.7	1.7	1.7
CD3+CD4+CD8a+FoxP3-CD25-	10.9	5.4	9.2
CD3+CD4+CD8a+FoxP3-CD25+	1.5	0.4	0.4
CD3+CD4+CD8a+FoxP3+CD25-	2.1	0.2	0.3
CD3+CD4+CD8a+FoxP3+CD25+	2.1	0.9	0.7

Table S7: Percent abundance of each cell type included in the cecal tissue network analysis (Figure 8). Data are from cecal tissue only. These data are an aggregate of all animals (combining the treatment groups).

Cell type	Percent total live single cells
CD3-CD4-CD8a+FoxP3-CD25+	0.359136239
CD3-CD4-CD8a+FoxP3-CD25-	4.962141485
CD3-CD4+CD8a+FoxP3-CD25-	0.197012368
CD3-CD4+CD8a-FoxP3-CD25-	0.501390276
CD3-CD4-CD8a-FoxP3-CD25+	1.534305545
CD3-CD4-CD8a-FoxP3+CD25-	0.982349568
CD3-CD4-CD8a-FoxP3-CD25-	26.50868465
CD3+CD4-CD8a+FoxP3-CD25+	2.905567331
CD3+CD4-CD8a+FoxP3+CD25+	0.109275604
CD3+CD4-CD8a+FoxP3-CD25-	32.87354777
CD3+CD4+CD8a+FoxP3-CD25+	1.014102178
CD3+CD4+CD8a+FoxP3+CD25+	1.35204093
CD3+CD4+CD8a+FoxP3+CD25-	1.323826585
CD3+CD4+CD8a+FoxP3-CD25-	7.13000692
CD3+CD4+CD8a-FoxP3-CD25+	0.87496106
CD3+CD4+CD8a-FoxP3+CD25+	0.426155555
CD3+CD4+CD8a-FoxP3+CD25-	0.308310558
CD3+CD4+CD8a-FoxP3-CD25-	10.95481137
CD3+CD4-CD8a-FoxP3-CD25+	0.354077344
CD3+CD4-CD8a-FoxP3+CD25-	0.153102137
CD3+CD4-CD8a-FoxP3-CD25-	4.824774696

Extended methods:

Flow Cytometry

Fresh cecal tissues were gently rinsed in PBS to remove as much digesta as possible and approximately 2 g of tissue was placed into a conical tube with 30 mL cRPMI media and stored on ice until returning to the laboratory for processing following an amended protocol previously described (Goodyear et al. 2014). For mucus dissolution, tissue was added to 30 mL solution of 5 mM DDT (cat: 15508-013, Invitrogen) and 2% FCS in calcium/magnesium-free Hank's balanced salt solution (HBSS), and incubated at 37°C in a MaxQ™ 2000 Benchtop Orbital Shaker (ThermoFisher) at 200 rpm for 20 min. Tissue was then transferred to 30 mL epithelial removal solution containing 5 mM EDTA and 2% FCS in HBSS, and a further 15 min incubation on the shaker as above, followed by a repeat of this step. The epithelial cells released during these 2 steps were collected by centrifugation as described below. The remaining lamina propria tissue was then transferred into a wash solution containing 10 mM HEPES (Sigma) in HBSS and incubated for 10 min on the shaker as above. Tissue was transferred to a C-tube (Miltenyi Biotec) with 14 mL enzyme digestion media containing 1% HEPES in HBBS, with 0.2 U/mL Liberase TM Research Grade (Roche Life Sciences), and 30 µg/mL DNase I (cat: D5025, Sigma Aldrich), mechanically minced with scissors, and subsequently processed on gentleMACS Octo Dissociator (Miltenyi Biotec) intestine setting for C-tubes before and after a 45 min incubation on the shaker as above. Enzymatically digested tissue was filtered through a sterile 4" x 4" gauze pad to collect released cells, and enzymes were inactivated by addition of 7 mL cRPMI. Cells were then run over a 40 uM mesh filter to remove any additional debris. Cells were centrifuged 450 x g for 8 min at 4°C, followed by resuspension of the pellet in a solution containing of 200 mM L-glutamine and 2% FCS, in HBSS. Epithelial cells released after incubation in EDTA solution were recovered via the same centrifugation were combined with cells from the lamina propria from the same respective animal. Approximately 10^6 cells were used for flow cytometric analysis.

Cells were first stained with Zombie Yellow Viability dye, followed by incubation with fluorescently-conjugated anti-porcine monoclonal antibodies purchased from BD Biosciences, San Jose, CA (except as noted), Antibodies used included anti-porcine CD3 (clone BB23-8E6-8C6, cat: 561477), CD4 (clone 74-12-4, cat: 559585), CD8α (clone 76-2-11, cat: 559584ID), CD25 (clone K231.3B2, Southern Biotech cat: 1070-19), and FOXP3 (clone FJK16s, cat: 48-5773-82). A cocktail of all surface marker antibodies was added to each cell suspension, followed by fixation and permeabilization for intracellular staining with anti-Foxp3 antibody using Intracellular Nuclear Staining Kit according to manufacturer's recommendations (Biolegend). Data was acquired on a BD LSRII machine and data was analyzed with FlowJo Software.

Immunohistochemical Staining (IHC)

Roughly 2 in x 2 in sections of fresh cecal tissues were stapled to a piece of dental wax and fixed in 10% buffered formalin for approximately 24 h prior to paraffin embedding using routine histological techniques. Paraffin blocks were sectioned to 4 µm on frosted microscope slides. Tissue sections were deparaffinized in xylene/ethanol (xylene 5 min x3, 100% EtOH 1 min x2, 95%, 85%, 70% EtOH for 1 min each, DI water wash 3 min, PBS) using a Leica ST5020

and outlined with a PAP pen (Invitrogen). All incubations were carried out in a StainTray™ humidity chamber containing 100mL tapwater at room temperature unless otherwise noted. Between each step, tissues were washed twice for 2 min each in PBST (1x PBS, 0.05% Tween20 [Sigma]).

IgA IHC staining protocol was adapted from that previously described by (Bianco et al. 2014). Briefly, endogenous peroxidase activity was quenched by incubation in 3% H_2O_2 in methanol for 30 min. Antigen retrieval was carried out by incubation in 1% Pronase E (cat: P6911, Sigma Aldrich) in PBS at 37°C for 10 min, followed by cooling for 10 min in PBS. Tissues were then incubated in Dual Endogenous Enzyme Blocker (Dako, Carpinteria, CA) for 10 mins to minimize non-specific staining. Tissues were incubated overnight (approx. 14-16 h) at 4°C with Goat Polyclonal Anti-Porcine IgA (cat: NB724, Novus Biologicals, Littleton, CO; dilution 1:60,000 in 1% Bovine Serum Albumin [BSA] in 1x PBS) primary antibody, followed by ImmPRESS™ HRP Anti-Goat Ig (Peroxidase) Polymer Detection Kit (cat: MP-7405, Vector Labs, Burlingame, CA) for 30 min.

To stain for CD3, heat-induced epitope retrieval was carried out by incubation in 10x sodium citrate buffer at pH 6.0, at 95°C for 20 min in a Biocare Medical Decloaker. After cooling, tissues were then incubated in Dual Endogenous Enzyme Blocker (Dako) for 10 min, followed by Universal Protein Blocker (Dako) for 20 min to minimize non-specific staining. Tissues were incubated with Polyclonal Rabbit Anti-Human IgG (A0452, Dako) for 1 h, followed by Dako EnVision+ system-HRP anti-rabbit polymer (K4002, Dako) for 30 min.

Antigen-antibody reactions were visualized with DAKO® Liquid DAB (3,3′diaminobenzidine tetrahydrochloride) + Substrate-Chromogen System (K3467, Dako) after a 3 min (CD3) or 5 min (IgA) incubation, and counterstained with Gills hematoxylin for 30 sec. Slides were dehydrated with a Leica ST5020 (90% EtOH/1min, 100% EtOH/1min x3, Propar 5 min x3) and coverslipped using a Leica CV5030 with Xylene Substitute Mountant (Shandon).

Positive and negative controls were utilized to assess the specificity of antibody staining. Sections of ileum were stained with primary antibody as positive controls. Negative controls were incubated in 1% BSA in 1x PBS without primary antibody. Additional 4 µm sections of cecal tissue from each animal were stained with hematoxylin and eosin (H&E) for evaluation of architectural integrity.

IHC-stained slides were subjectively evaluated at 10X and 40X using an EVOS XL Core Cell Imaging System to confirm the specificity of staining. Slides were then scanned into Spectrum Version 11.2.0.780 (Aperio Technologies, Inc.) and computer aided morphometry with Aperio ImageScope was utilized for annotation and to quantify cell populations. Cell counts were obtained using a nuclear algorithm on Aperio ScanScope Software tuned to the level of staining. Cell counts are reported as cells/mm^2. Photomicrographs were taken with a Nikon DS-Ri1 digital camera on a Nikon Eclipse Ni-E microscope, and using NIS-Elements Imaging Software.

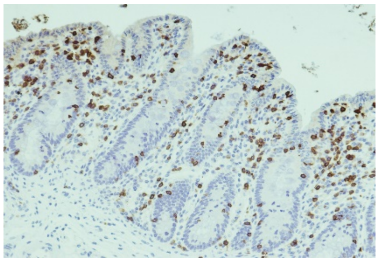

Figure S4A. CD3$^+$ IHC staining, cecum, CON group, 20X.

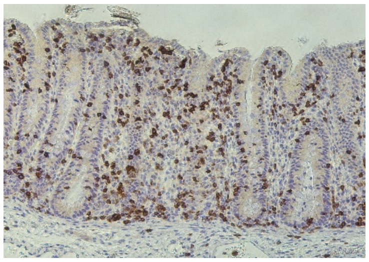

Figure S4B. CD3$^+$ IHC staining, cecum, RS group, 20X.

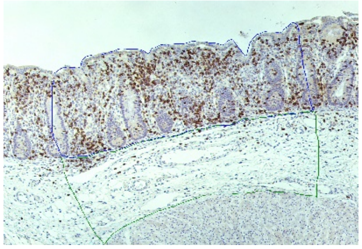

Figure S4E. Annotation dividing mucosa and submucosa in CD3$^+$ IHC-stained tissues, cecum, RS group, 10X.

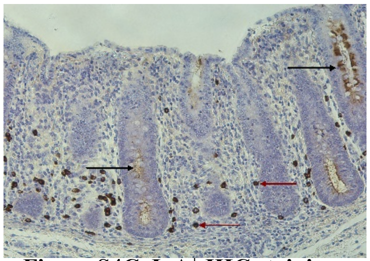

Figure S4C. IgA$^+$ IHC staining, cecum, CON group, 20X. IgA-secreting cells are indicated by red arrows, sIgA is indicated by black arrows.

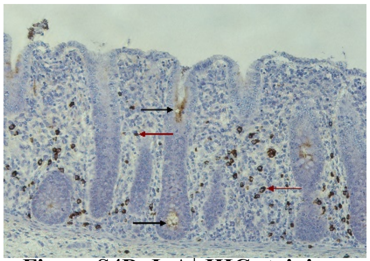

Figure S4D. IgA$^+$ IHC staining, cecum, RS group, 20X. IgA-secreting cells are indicated by red arrows, sIgA is indicated by black arrows.

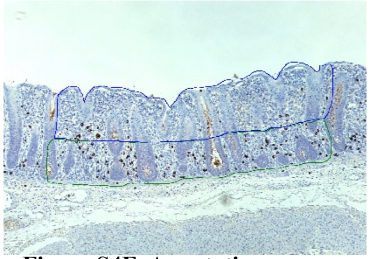

Figure S4F. Annotation dividing apical and basal mucosa in IgA IHC-stained tissues, cecum, RS group, 10X.

.
